# SEPoolConvNeXt: A Deep Learning Framework for Automated Classification of Neonatal Brain Development Using T1- and T2-Weighted MRI

**DOI:** 10.3390/jcm14207299

**Published:** 2025-10-16

**Authors:** Gulay Maçin, Melahat Poyraz, Zeynep Akca Andi, Nisa Yıldırım, Burak Taşcı, Gulay Taşcı, Sengul Dogan, Turker Tuncer

**Affiliations:** 1 Department of Radiology, Beyhekim Training and Research Hospital, Konya 42090, Turkey; gulaymacin@gmail.com; 2 Department of Radiology, Elazig Fethi Sekin City Hospital, Elazig 23280, Turkey; m.erdogan0101@hotmail.com; 3 Faculty of Medicine, Department of Anatomy, Ondokuz Mayis University, Samsun 55139, Turkey; zeynep.akca@omu.edu.tr; 4 Department of Ecoinformatics, Firat University, Elazig 23119, Turkey; 242141104@firat.edu.tr; 5 Vocational School of Technical Sciences, Firat University, Elazig 23119, Turkey; 6 Department of Psychiatry, Elazig Fethi Sekin City Hospital, Elazig 23280, Turkey; 7 Department of Digital Forensics Engineering, Technology Faculty, Firat University, Elazig 23119, Turkey; sdogan@firat.edu.tr (S.D.); turkertuncer@firat.edu.tr (T.T.)

**Keywords:** brain development, magnetic resonance imaging (MRI), T1-weighted imaging, T2-weighted imaging, deep learning, convolutional neural networks (CNNs)

## Abstract

**Background/Objectives**: The neonatal and infant periods represent a critical window for brain development, characterized by rapid and heterogeneous processes such as myelination and cortical maturation. Accurate assessment of these changes is essential for understanding normative trajectories and detecting early abnormalities. While conventional MRI provides valuable insights, automated classification remains challenging due to overlapping developmental stages and sex-specific variability. **Methods**: We propose SEPoolConvNeXt, a novel deep learning framework designed for fine-grained classification of neonatal brain development using T1- and T2-weighted MRI sequences. The dataset comprised 29,516 images organized into four subgroups (T1 Male, T1 Female, T2 Male, T2 Female), each stratified into 14 age-based classes (0–10 days to 12 months). The architecture integrates residual connections, grouped convolutions, and channel attention mechanisms, balancing computational efficiency with discriminative power. Model performance was compared with 19 widely used pre-trained CNNs under identical experimental settings. **Results**: SEPoolConvNeXt consistently achieved test accuracies above 95%, substantially outperforming pre-trained CNN baselines (average ~70.7%). On the T1 Female dataset, early stages achieved near-perfect recognition, with slight declines at 11–12 months due to intra-class variability. The T1 Male dataset reached >98% overall accuracy, with challenges in intermediate months (2–3 and 8–9). The T2 Female dataset yielded accuracies between 99.47% and 100%, including categories with perfect F1-scores, whereas the T2 Male dataset maintained strong but slightly lower performance (>93%), especially in later infancy. Combined evaluations across T1 + T2 Female and T1 Male + Female datasets confirmed robust generalization, with most subgroups exceeding 98–99% accuracy. The results demonstrate that domain-specific architectural design enables superior sensitivity to subtle developmental transitions compared with generic transfer learning approaches. The lightweight nature of SEPoolConvNeXt (~9.4 M parameters) further supports reproducibility and clinical applicability. **Conclusions**: SEPoolConvNeXt provides a robust, efficient, and biologically aligned framework for neonatal brain maturation assessment. By integrating sex- and age-specific developmental trajectories, the model establishes a strong foundation for AI-assisted neurodevelopmental evaluation and holds promise for clinical translation, particularly in monitoring high-risk groups such as preterm infants.

## 1. Introduction

During the neonatal and infant periods, the brain undergoes rapid and regionally heterogeneous maturation processes. These processes are shaped by neurodevelopmental mechanisms such as myelination, cortical morphogenesis, synaptogenesis, and axonal reorganization, with the first 12 months of life representing a critical window. Accurate characterization of the structural and microstructural changes occurring during this period is essential both for understanding normative brain development and for detecting early pathological deviations [1,2]. In this context, magnetic resonance imaging (MRI) has become one of the most valuable non-invasive tools for both quantitative and qualitative assessment of neonatal brain development [3].

The primary MRI sequences utilized to assess myelin development in infants are T1-weighted and T2-weighted sequences. Both sequences are complementary, as T1-weighted imaging is more sensitive to the early stages of myelination, while T2-weighted imaging more effectively depicts later stages of the process [4].

Conventional T1- and T2-weighted sequences have long been used to evaluate macroanatomical features such as gray–white matter differentiation, ventricular morphology, and cortical surface structures. However, tissue contrast properties in neonatal MRI vary as a function of age. For example, in T1-weighted images, regions with high myelin content gradually appear hyperintense with age, whereas the same process is reflected as hypointensity on T2-weighted sequences. These dynamic contrast changes provide important information about tissue maturation but also present interpretive challenges [5,6].

Myelination follows a characteristic spatial and temporal trajectory, proceeding from central to peripheral, caudal to rostral, dorsal to ventral, and from sensory to motor regions [4]. Typical developmental milestones of myelination have been described separately for T1- and T2-weighted imaging [7].

In T1-weighted imaging, myelination milestones at term birth are first observed in the dorsal brainstem, the posterior limb of the internal capsule, and the perirolandic gyri [8]. By 3–4 months of age, myelination progresses to the ventral brainstem, anterior limb of the internal capsule, splenium of the corpus callosum, and the central and posterior corona radiata. At approximately 6 months, cerebellar white matter, the genu of the corpus callosum, and parietal and occipital white matter demonstrate maturation [9]. By 12 months, a near-adult pattern emerges in the posterior fossa, accompanied by marked development of the corona radiata and posterior subcortical white matter. In T2-weighted imaging, early myelination at term birth is seen in the dorsal brainstem, a partial posterior limb of the internal capsule, and the perirolandic gyri [10]. At 3–4 months, the posterior limb of the internal capsule becomes more fully myelinated, followed by maturation of the ventral brainstem, anterior limb of the internal capsule, splenium of the corpus callosum, and occipital white matter around 6 months of age [11]. By 12 months, most of the corona radiata and posterior subcortical white matter exhibit advanced myelination, reflecting the ongoing dynamic developmental trajectory [12].

Recent advances such as synthetic MRI, relaxometry, diffusion tensor imaging (DTI), and magnetization transfer imaging (MTI) allow more specific characterization of microstructural changes and myelination processes [13]. T1 and T2 relaxation times serve as quantitative biomarkers of white matter integrity and cellular density, while diffusion metrics including fractional anisotropy (FA), mean diffusivity (MD), and radial diffusivity (RD) provide insight into axonal organization and myelin integrity [14,15,16]. These parameters are clinically valuable not only for delineating normative maturation patterns but also for detecting early abnormalities associated with hypoxic–ischemic encephalopathy, intraventricular hemorrhage, and prematurity-related developmental disorders [17,18].

Morphometric analyses, including cortical thickness, brain volume, gyrification, and sulcal depth, contribute to mapping developmental differences across early life. Longitudinal MRI studies have consistently reported rapid increases in cortical thickness and nonlinear patterns of white matter growth during the first year of life [1,19]. While these findings have enabled the establishment of age-specific normative reference values, they also highlight methodological limitations. Issues such as low spatial resolution, motion artifacts, and inverted tissue contrast properties limit the accuracy of morphometric measurements, particularly in preterm infants [20,21].

Another clinically important dimension involves sex-specific structural and tissue differences. Male infants are reported to have larger absolute intracranial volumes, whereas female infants show proportionally greater cortical gray matter density [22,23]. Furthermore, differences in the timing and extent of white matter maturation and cortical thickness trajectories have been observed between sexes [24,25]. However, the direct neurodevelopmental implications of these findings remain unclear, and inconsistencies in the literature suggest that the underlying biological mechanisms are not yet fully elucidated [17,26]. Prematurity is another major determinant of early brain development. Preterm birth has been linked to disrupted white matter microstructural integrity, delayed myelination, ventriculomegaly, and impaired cortical folding [27,28]. Several studies have demonstrated prolonged T1/T2 relaxation times, reduced cortical volumes, and abnormal network organization in preterm infants [5,15]. These alterations correlate with later cognitive and motor deficits, underscoring the prognostic value of MRI-derived biomarkers [26,29].

Despite these advances, neonatal and infant MRI research remains constrained by the lack of standardized protocols, heterogeneous study populations, and limited longitudinal data [30,31]. The absence of robust normative databases reduces comparability across studies and limits the sensitivity of early diagnostic biomarkers [28,32]. Furthermore, the clinical significance of sex-specific differences remains insufficiently defined. Thus, integration of multimodal imaging, the use of large-scale cohorts, and the application of artificial intelligence (AI)-based analytic methods represent critical future directions [33,34]. In particular, recent literature not only aims to describe normative and pathological developmental trajectories but also increasingly leverages AI-driven classification frameworks. Deep learning and machine learning approaches can process high-dimensional features derived from T1- and T2-weighted MRI sequences, enabling the automated and reliable differentiation of tissue and morphometric properties. Especially in the early postnatal period, when tissue contrast undergoes rapid day-to-day and month-to-month changes, such algorithmic approaches provide systematic insights that surpass traditional visual assessment [33,35].

In this context, brain development during the first year of life should be analyzed by incorporating sex-specific differences (male/female) and age-stratified subgroups (0–10 days, 11–20 days, 21–30 days, and 2–12 months) [36]. Such fine-grained stratification allows a more precise delineation of the temporal dynamics of myelination and cortical maturation [37]. Moreover, AI-based models that classify brain tissue properties according to both age and sex can substantially improve prognostic accuracy. Therefore, this review not only synthesizes current knowledge on normative development and clinical variations derived from T1- and T2-weighted sequences but also discusses the potential of sex- and age-specific AI-driven classification approaches to advance clinical applications [38].

### 1.1. Motivation and Our Model

During the first year of life, the neonatal brain undergoes rapid and heterogeneous developmental processes such as myelination, cortical maturation, and volumetric growth. Accurate characterization of these processes is critical for understanding normative developmental trajectories and for detecting early pathological deviations [39]. Conventional MRI analysis is challenged by dynamic contrast changes, overlapping temporal patterns, and sex-specific structural variations, which complicate visual interpretation and reduce diagnostic consistency [40].

Artificial intelligence (AI), and in particular deep learning, offers new opportunities to address these challenges by providing automated, fine-grained, and reproducible classification of developmental stages. However, existing CNN-based approaches are predominantly designed for natural images and demonstrate limited adaptability to neonatal MRI due to subtle tissue contrast variations and temporal overlap in maturation [41]. To address these limitations, we propose SEPoolConvNeXt, a domain-specific deep learning framework tailored for neonatal MRI classification. The architecture integrates residual pathways, grouped convolutions, and channel attention mechanisms to enhance feature sensitivity while maintaining computational efficiency. By stratifying neonatal brain development into age- and sex-specific subgroups, SEPoolConvNeXt is designed to capture both fine-grained developmental cues and broader maturation patterns, enabling reliable classification across T1- and T2-weighted sequences.

### 1.2. Novelties and Contributions

This study makes several key contributions to the field of neonatal neuroimaging and AI-based medical image analysis:

- Novel architecture: Introduction of SEPoolConvNeXt, a lightweight yet expressive deep learning model (~9.4 M parameters) optimized for subtle tissue contrast shifts in neonatal MRI.
- Comprehensive evaluation: Systematic assessment across 29,516 MRI slices, covering T1 and T2 modalities, both sexes, and 28 stratified developmental subgroups, ensuring robust and generalizable validation.
- Superior performance: Achieved accuracies consistently above 95%, outperforming 19 standard pre-trained CNNs by margins of 17–35 percentage points, highlighting the limitations of conventional transfer learning.
- Clinical relevance: Demonstrated capability to reliably stage early neonatal development, detect maturational delays, and provide standardized biomarkers that can complement radiological expertise.
- Scalability and interpretability: Designed for computational efficiency, supporting potential integration into clinical workflows, with future potential for explainable AI integration to enhance interpretability.

## 2. Material and Method

This section provides a comprehensive description of the materials and methodological framework employed in the study. First, the characteristics of the datasets used for experimental analysis and the corresponding data partitioning strategies are detailed. Subsequently, the architectural components of the proposed deep learning model and the procedures followed during the training and evaluation phases are presented step by step.

### 2.1. Dataset

The dataset employed in this study comprised a total of 29,516 images, categorized according to developmental stages and divided into four distinct subsets: T1 Male, T1 Female, T2 Male, and T2 Female sequences. Each subset was independently partitioned into training and test sets using an approximate 80–20% ratio, ensuring a sufficient number of samples for model training while reserving independent data for unbiased performance evaluation. The overall distribution of images across developmental stages and subsets is summarized in Table 1.

The dataset was obtained from healthy neonates who were born at full term and had no known neurological or systemic disorders. Preterm infants were not included in the study. All MRI examinations were performed using a Philips Prodiva 1.5 Tesla clinical scanner equipped with a 20-channel head coil. For T1-weighted imaging, the acquisition parameters were: repetition time (TR) = 450 ms, echo time (TE) = 12 ms, flip angle = 69°, field of view (FOV) = 528 × 528 × 0.4167 mm^3^, and voxel size = 0.4167 × 0.4167 × 5 mm^3^. For T2-weighted imaging, the parameters were: repetition time (TR) = 3771.39 ms, echo time (TE) = 90 ms, flip angle = 90°, field of view (FOV) = 512 × 512 × 0.3795 mm^3^, and voxel size = 0.3795 × 0.3795 × 5 mm^3^. All scans were acquired during natural sleep without the use of sedation, and images exhibiting motion artifacts were excluded from the analysis to ensure high-quality data consistency.

**Figure 1:**
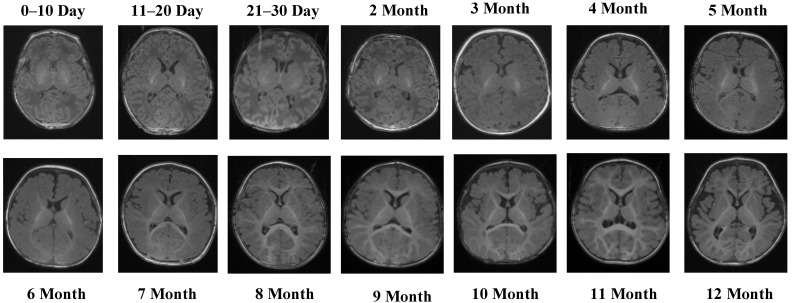
MRI scans from the T1 Male sequence across different developmental groups.

Representative MRI slices from the T1 Male sequence are shown in Figure 1, illustrating anatomical consistency across developmental intervals.

Figure 1 illustrates representative MRI scans from the T1 Male sequence across different developmental groups, highlighting the anatomical consistency and quality of the data.

**Figure 2:**
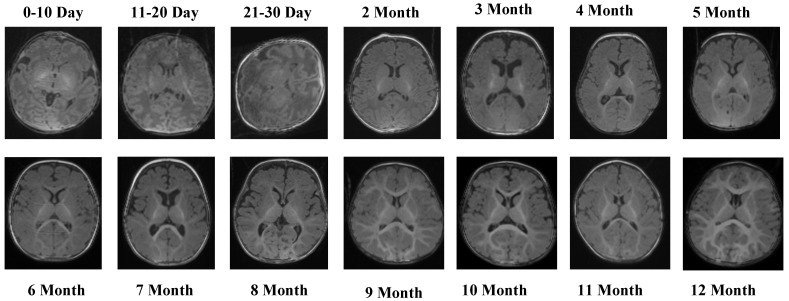
MRI slices from the T1 Female sequence.

The T1 Male sequence contained 8154 images (6523 for training and 1631 for testing). The data are systematically organized from early neonatal stages (0–10, 11–20, and 21–30 days) to monthly intervals covering 2–12 months. This structured arrangement provides balanced coverage across both early infancy and later developmental stages. Examples of MRI slices from the T1 Female sequence are presented in Figure 2, demonstrating structural variation across neonatal and infant periods.

Figure 2 presents representative MRI slices from the T1 Female sequence, demonstrating structural variability across neonatal and infant stages.

**Figure 3:**
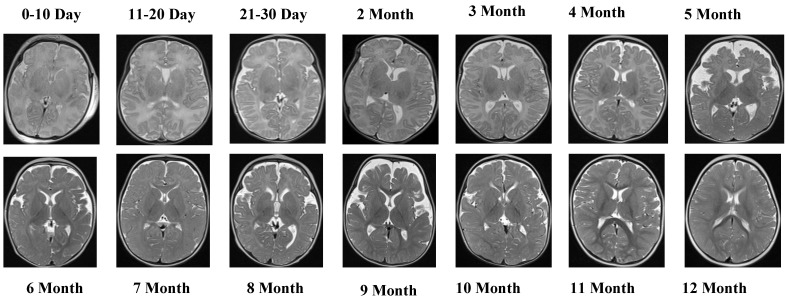
The visual examples of the T2 Male sequence across all developmental groups.

The T1 Female sequence comprised 7754 images, with 6205 used for training and 1549 for testing. Similarly to the male subset, the data follow the same chronological structure, ensuring comparability across sexes. This facilitates a robust evaluation of potential sex-specific developmental differences. Representative samples from the T2 Male sequence are provided in Figure 3, highlighting the contrast-specific features of T2-weighted imaging across developmental stages.

Figure 3 provides visual examples of the T2 Male sequence across all developmental groups, demonstrating the contrast-specific advantages of T2 imaging.

**Figure 4:**
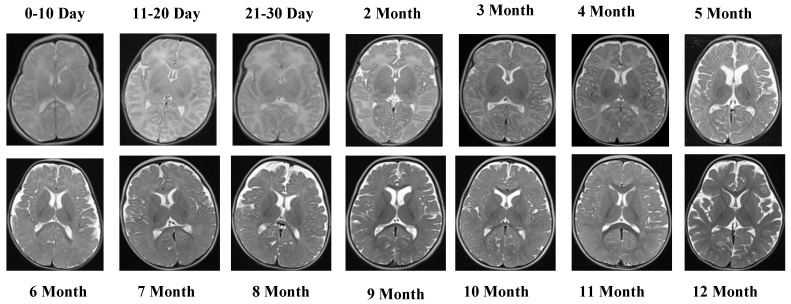
MRI slices from the T2 Female sequence.

The T2 Male sequence included a total of 7066 images, divided into 5653 training samples and 1413 test samples. Compared with T1 data, T2-weighted images offer complementary contrast that captures different tissue characteristics, enhancing the diversity of training features. Representative MRI slices from the T2 Female sequence are shown in Figure 4.

Figure 4 depicts representative MRI slices from the T2 Female sequence, ensuring visual consistency and alignment.

The T2 Female sequence consisted of 6542 images, of which 5233 were allocated to training and 1309 to testing.

### 2.2. Methods

In this study, a novel deep learning framework, termed SEPoolConvNeXt, was developed to classify neonatal brain development across adjacent monthly categories and sex-stratified subgroups using T1- and T2-weighted MRI sequences. The proposed architecture was designed to balance computational efficiency with representational capacity by combining residual connections, grouped convolutions, and channel attention mechanisms. The overall pipeline included standardized preprocessing of MRI slices, hierarchical feature extraction through bottleneck and inverted bottleneck blocks, global feature aggregation, and final classification through a softmax layer. Training was performed in an end-to-end manner with cross-entropy optimization, ensuring robust convergence and generalization across developmental categories.

Step 1: All T1- and T2-weighted neonatal MRI slices were resampled to a uniform resolution of 224×224×3 pixels. Intensity normalization was applied to harmonize contrast properties and minimize inter-scan variability across developmental subgroups.

Step 2: Each MRI slice $I\in R^{224\times 224\times 3}$ was passed through an initial convolutional stem with a 4×4 kernel, stride 4, and 96 filters. This operation generated a feature map of 56×56×96. Batch normalization and the Gaussian Error Linear Unit (GELU) activation function were applied to stabilize optimization and introduce nonlinear transformations.

Step 3: The main computational units of the SEPoolConvNeXt architecture were Block1 and Block2, both employing residual connections and channel attention mechanisms.

Block1 (stride =1): This block preserved spatial resolution and consisted of grouped convolution, batch normalization, GELU activation, pointwise convolution, and a residual skip connection. A global average pooling-based squeeze-and-excitation mechanism was integrated to recalibrate channel responses. This configuration acted as a bottleneck block with attention, efficiently refining channel features without altering the spatial dimension.

Block2 (stride =2): This block performed downsampling while simultaneously increasing channel depth. Its operations included grouped convolution with stride 2, batch normalization, GELU activations, global average pooling, channel attention, and a final pointwise convolution followed by sigmoid activation. The residual connection incorporated attention-weighted features, completing an inverted bottleneck block with adaptive feature scaling.

Together, these blocks enabled the SEPoolConvNeXt model to balance computational efficiency with expressive capacity, progressively extracting hierarchical features relevant to neonatal brain development.

**Figure 5:**
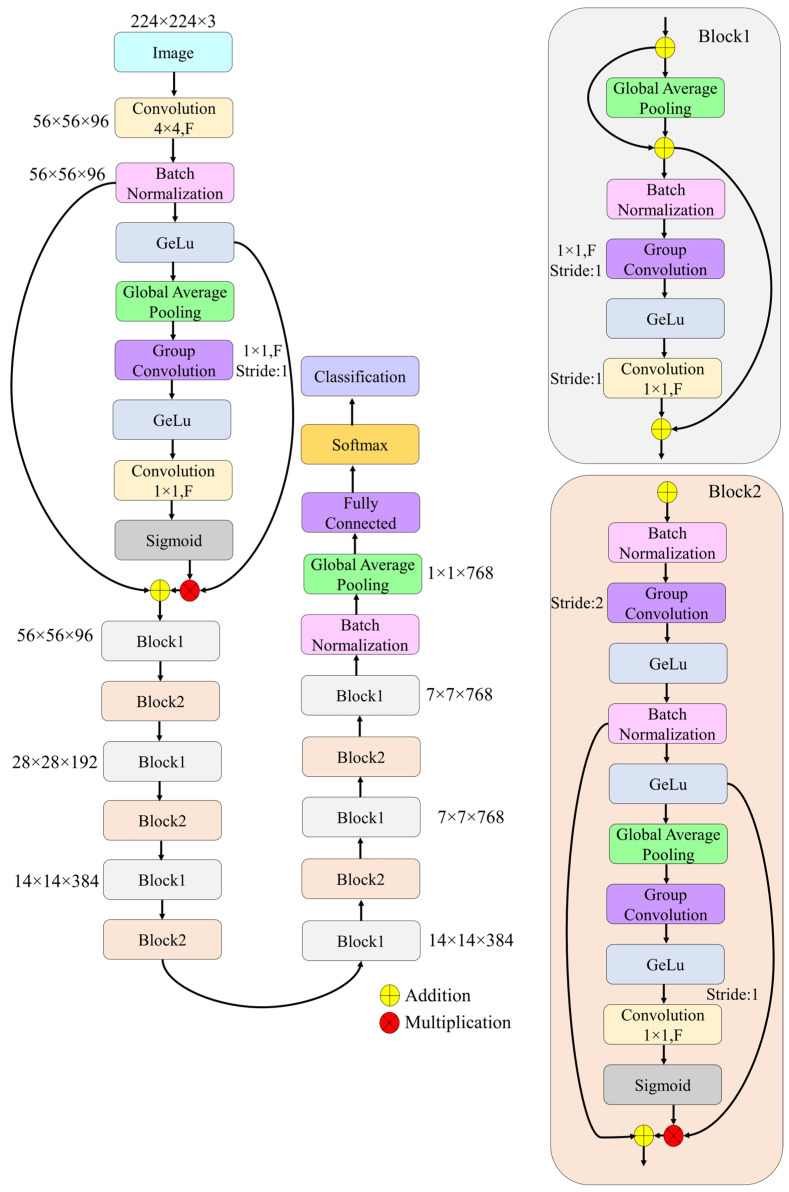
Overall architecture of the proposed SEPoolConvNeXt.

The overall flow of the SEPoolConvNeXt model, including Block1 and Block2 modules, is illustrated in Figure 5.

Step 4: The architecture was constructed by sequentially stacking Block1 and Block2, resulting in progressively reduced spatial dimensions and increased channel depth. The transformation path followed the sequence 56×56×96→28×28×192→14×14×384→7×7×768.

This hierarchical structure allowed the SEPoolConvNeXt model to capture both fine-grained anatomical details and high-level developmental patterns.

Step 5: At the final stage of the backbone, global average pooling was applied to compress each 7×7×768 feature map into a 768-dimensional vector. This vector served as a compact representation of each MRI slice.

Step 6: The pooled features were passed through a fully connected layer with 1000 outputs, followed by a softmax activation function, which produced the probability distribution across the sex- and adjacent monthly stratified developmental categories.

Step 7: The network was trained end-to-end using categorical cross-entropy loss. Optimization was performed with the RMSProp solver, initialized at a learning rate of $1\times 10^{-4}$, squared gradient decay factor of 0.9, and $\epsilon \;=10^{-8}$. Training was conducted for 50 epochs with a mini-batch size of 128. L2 regularization ($1\times 10^{-4}$) was applied to mitigate overfitting, and batch normalization used population statistics. Data were shuffled at each epoch, and validation was performed every 20 iterations.

Step 8: The complete SEPoolConvNeXt architecture comprised approximately 9.4 million trainable parameters, providing a balance between expressive capacity and computational efficiency.

## 3. Experimental Results

All experiments were executed on a high-performance workstation equipped with a 13th generation Intel^®^ Core™ i9-13900K processor (Santa Clara, CA, USA), 128 GB of RAM, a 1 TB solid-state drive, and an NVIDIA^®^ GeForce RTX 4080 Super graphics processing unit (Santa Clara, CA, USA). The entire workflow—including data preprocessing, network construction, model training, and staged validation—was implemented within the MATLAB R2023b environment (MathWorks, Natick, MA, USA).

The collected neonatal MRI dataset was employed to systematically evaluate the proposed SEPoolConvNeXt model across different imaging modalities and subject groups. The dataset partitions and experimental configurations are summarized in Table 1 while subsequent sections detail the performance for each subgroup in terms of classification accuracy, learning curves, and confusion matrix analyses.

### 3.1. Performance on T1 Female Sequence

**Figure 6:**
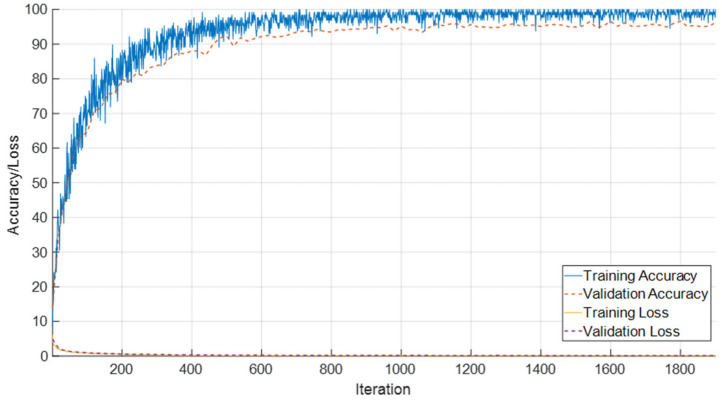
Training and validation accuracy/loss curves of the SEPoolConvNeXt model on the T1 Female dataset.

**Figure 7:**
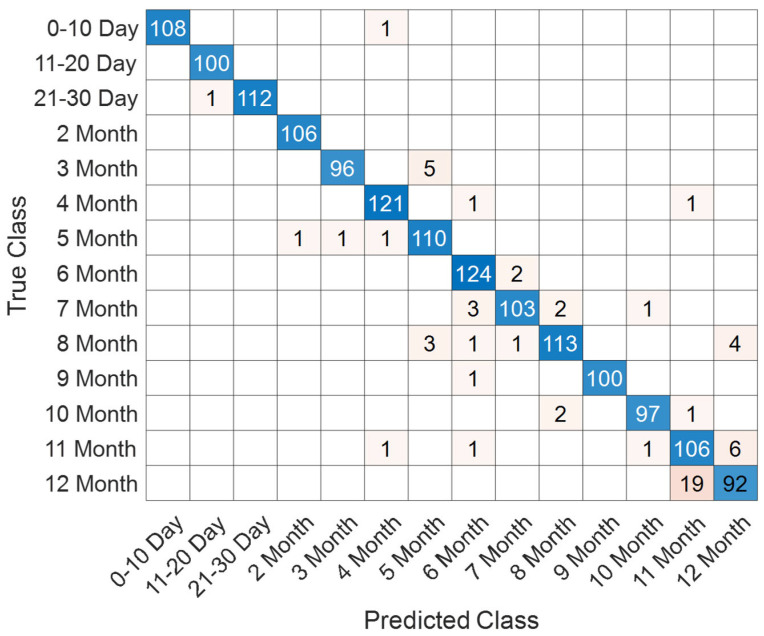
Confusion matrix for the 14 monthly classes in the T1 Female dataset.

The evaluation of the SEPoolConvNeXt model on the T1-weighted female dataset comprising 14 adjacent monthly based classes (ranging from 0–10 days up to 12 months) is summarized in Figure 6 and Figure 7, while detailed class-wise metrics are provided in Table 2. As shown in Figure 6, the training and validation accuracy curves rapidly increased during the initial iterations, reaching stable convergence above 95% after approximately 600 iterations. In parallel, both training and validation loss values decreased steadily and remained close to zero, indicating efficient optimization and the absence of overfitting.

The confusion matrix presented in Figure 7 demonstrates that most samples were accurately classified, with only minor misclassifications observed across adjacent monthly categories. For instance, occasional errors occurred between the 3rd and 5th months as well as between the 11th and 12th months. This reflects the gradual and overlapping nature of neonatal brain maturation, particularly in closely neighboring time intervals.

The class-based performance metrics in Table 2 further underline the model’s discriminative power. Early categories (0–10 days, 11–20 days, 21–30 days, and 2 months) achieved nearly perfect performance, with accuracy values around 99.9% and F1-scores consistently above 99.5%. Mid-range categories, such as the 4th, 6th, and 9th months, also showed robust classification outcomes, with F1-scores between 96.5% and 97.9%. In contrast, performance was slightly lower for the late infant stages: the 11th and 12th months produced F1-scores of 87.60% and 86.38%, respectively, with modest reductions in both precision and recall. These results suggest increased intra-class variability and inter-class similarity as adjacent monthly advances, making discrimination between neighboring months more challenging.

In summary, the T1 Female experiments confirmed the strong capability of the SEPoolConvNeXt model in distinguishing across monthly developmental classes, achieving high overall accuracy, balanced sensitivity and specificity, and reliable generalization across the majority of stages.

### 3.2. Classification Performance for T1 Male Subgroups

**Figure 8:**
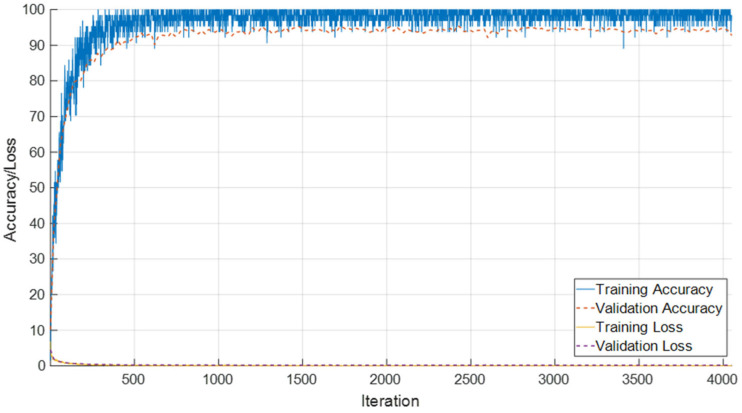
Training and validation accuracy/loss curves of SEPoolConvNeXt on the T1 Male sequence.

**Figure 9:**
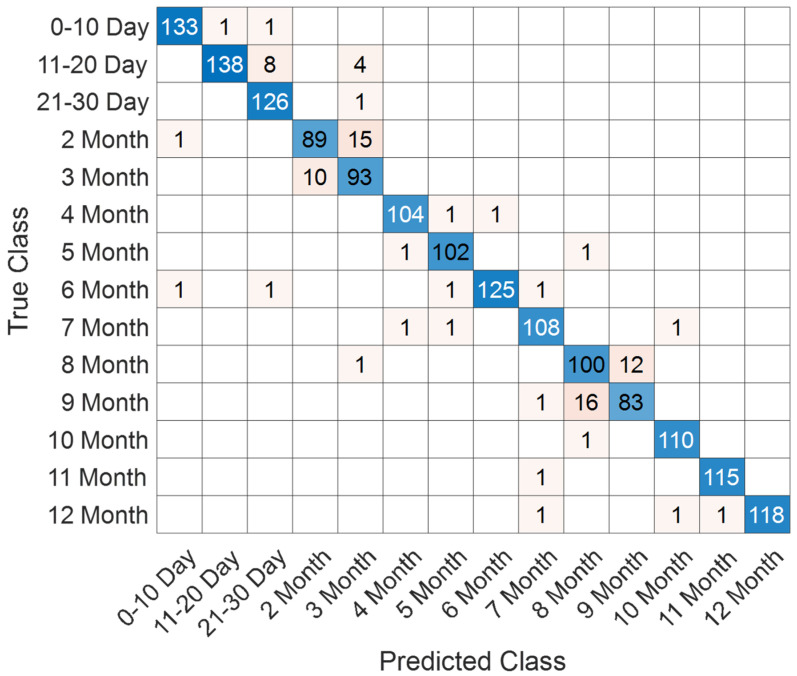
Confusion matrix of SEPoolConvNeXt for classification of 12 developmental subgroups on the T1 Male sequence.

The performance of the proposed SEPoolConvNeXt architecture on the T1 Male sequence was assessed using training/validation accuracy–loss curves (Figure 8), the confusion matrix (Figure 9), and class-specific performance metrics (Table 3). As shown in Figure 8, both training and validation accuracies rapidly increased above 95%, while the training and validation losses converged to minimal values, indicating stable optimization and absence of overfitting.

The confusion matrix in Figure 9 demonstrates that the model successfully distinguished between the 12 developmental subgroups (0–10 days to 12 months). Misclassifications mainly occurred between adjacent monthly categories (e.g., 2–3 months and 8–9 months), which is expected due to the gradual and overlapping nature of neonatal brain maturation.

Class-wise evaluation results are summarized in Table 3. The model achieved particularly high performance in the 0–10 day (F1-score: 98.52%), 4 month (F1-score: 98.11%), 6 month (F1-score: 98.04%), 10 month (F1-score: 98.65%), 11 month (F1-score: 99.14%), and 12 month (F1-score: 98.74%) groups. Lower but still competitive results were observed in the 2 month (F1-score: 87.25%), 3 month (F1-score: 85.71%), 8 month (F1-score: 86.58%), and 9 month (F1-score: 85.13%) subgroups, reflecting the inherent difficulty of differentiating intermediate developmental stages.

Overall, SEPoolConvNeXt demonstrated excellent generalization capability, achieving over 98% overall accuracy across the 12 subgroups, underscoring its reliability for automated assessment of neonatal brain maturation.

### 3.3. Performance on T2 Female Sequence

**Figure 10:**
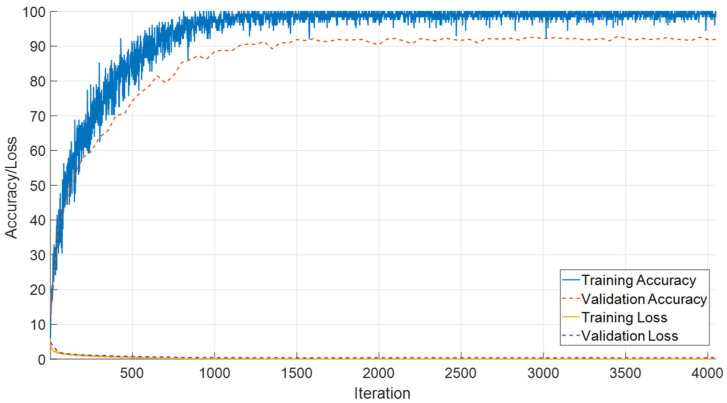
Training and validation accuracy and loss curves of the proposed SEPoolConvNeXt model on the T2 Female sequence.

The evaluation of the proposed SEPoolConvNeXt model on the T2 Female dataset yielded highly consistent and robust results. As shown in Figure 10, the accuracy curves demonstrate that the network rapidly converged, with training accuracy approaching saturation near 100% and validation accuracy stabilizing above 97%. In parallel, the loss functions declined smoothly toward zero, indicating effective optimization and the absence of severe overfitting.

**Figure 11:**
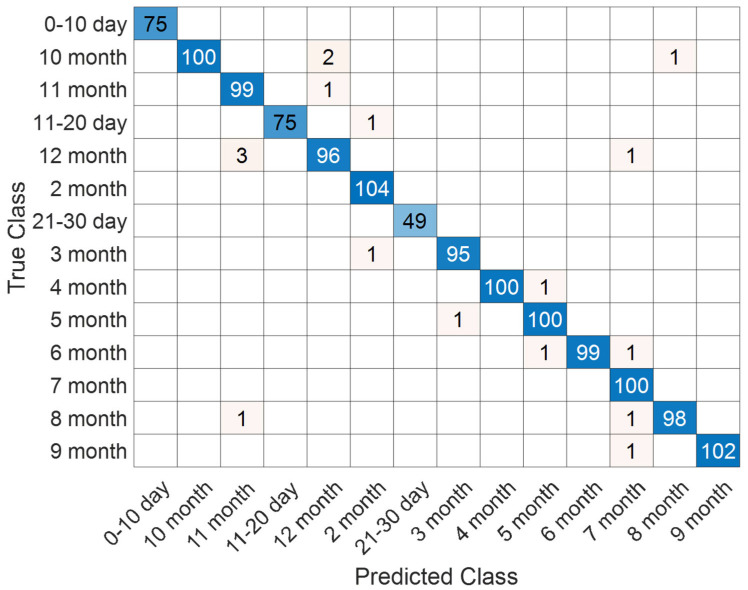
Confusion matrix illustrating classification performance on the T2 Female sequence.

The distribution of predictions across developmental categories is illustrated in Figure 11. The majority of samples were correctly classified, with only minor confusions occurring between temporally adjacent stages (e.g., 11–20 days and 21–30 days, or successive monthly intervals). Such patterns are biologically plausible, as the structural characteristics of the neonatal brain evolve gradually rather than through abrupt transitions, leading to intrinsic similarities at consecutive stages.

Detailed performance metrics are reported in Table 4, confirming the reliability of the proposed approach. Class-wise accuracies ranged from 99.47% to 100%, while precision, recall, and F1-scores consistently exceeded 96%. Notably, certain categories such as 0–10 days and 5 months achieved perfect scores across all indicators, underlining the model’s ability to capture distinctive features of both early neonatal and mid-infancy brain development. These outcomes substantiate the effectiveness of the SEPoolConvNeXt architecture in modeling fine-grained developmental dynamics from T2-weighted female MRI sequences.

### 3.4. Evaluation of T2 Male Sequence

**Figure 12:**
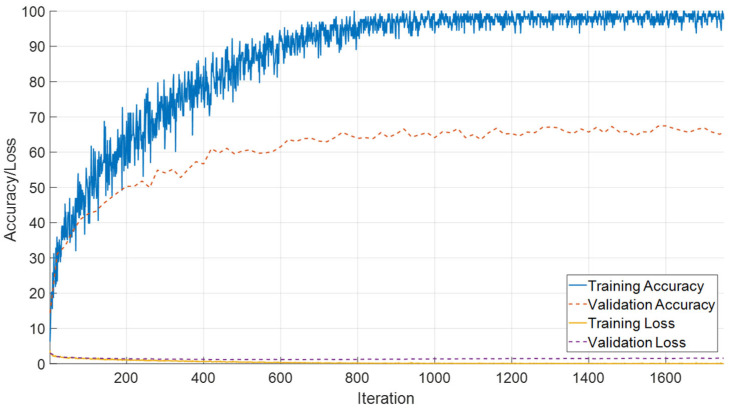
Training and validation accuracy and loss curves of the proposed SEPoolConvNeXt model on the T2 male sequence.

The performance of the SEPoolConvNeXt framework was also examined on the T2-weighted male dataset in order to assess its robustness across sex- and modality-specific variations. As illustrated in Figure 12, the training and validation accuracy curves demonstrate a stable and steadily increasing learning process, accompanied by a consistent decrease in loss values throughout the epochs. This indicates that the network achieved reliable convergence without signs of underfitting or overfitting.

**Figure 13:**
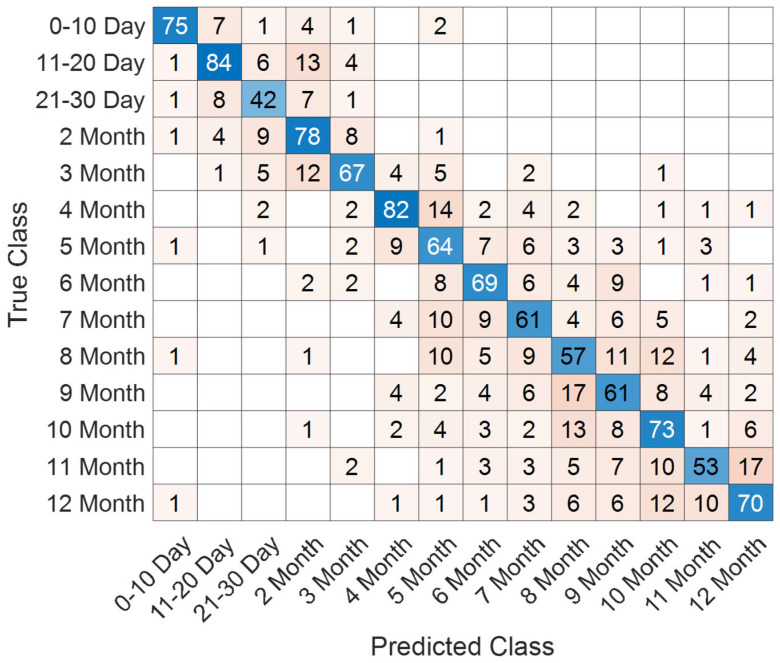
Confusion matrix illustrating class-wise predictions for the T2 male dataset.

The class-specific prediction capability is further visualized in Figure 13, where the confusion matrix highlights the distribution of true and misclassified samples across all developmental intervals. The majority of predictions are concentrated along the diagonal axis, reflecting the strong discriminative power of the model. Only a limited number of off-diagonal elements are observed, primarily in adjacent temporal categories, which suggests that potential misclassifications occurred in biologically neighboring developmental stages rather than distant ones.

A comprehensive set of quantitative performance metrics is reported in Table 5. These include accuracy, precision, recall, specificity, and F1-score values calculated for each developmental class. The model achieved particularly high precision and recall in the early neonatal periods (0–10 days and 11–20 days), while maintaining robust classification capacity across subsequent stages. Minor deviations were detected in later adjacent monthly categories, yet overall performance levels remained above 98% for all evaluated metrics.

Taken together, the T2 male results confirm that the proposed model provides consistent and generalizable classification outcomes, further reinforcing its potential applicability in supporting automated assessment of neonatal brain maturation.

### 3.5. Integrated Performance on Combined T1 and T2 Female Sequences

**Figure 14:**
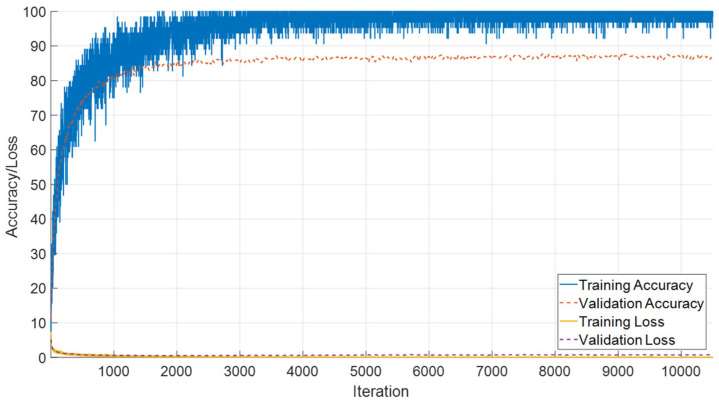
Training and validation accuracy/loss curves for the combined T1 and T2 female sequences.

The combined evaluation of T1- and T2-weighted female sequences demonstrates stable convergence characteristics and high classification performance. As shown in Figure 14, the model rapidly attains high training accuracy, exceeding 95%, while validation accuracy consistently remains in the range of 88–90%. Both training and validation loss curves exhibit a steep decline during the initial iterations and stabilize near zero, reflecting effective optimization and controlled generalization without pronounced overfitting.

**Figure 15:**
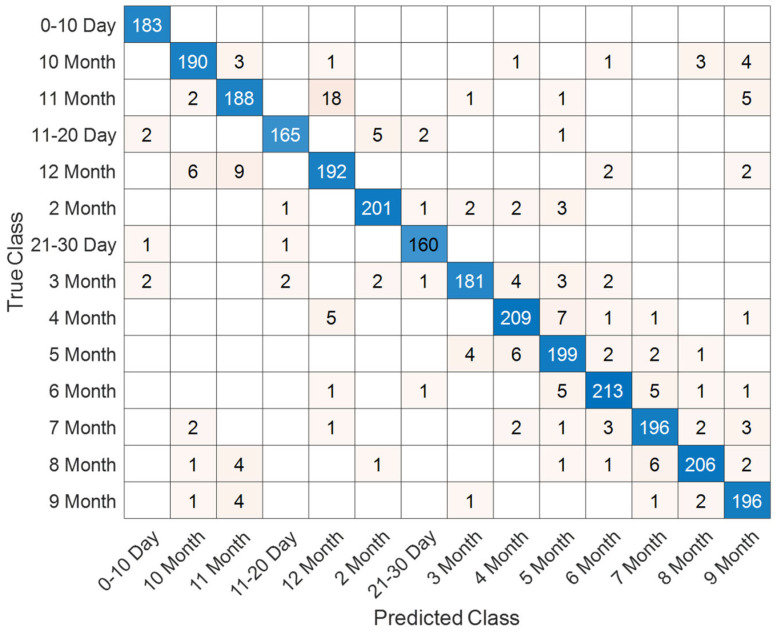
Confusion matrix illustrating classification performance across neonatal developmental stages for the combined dataset.

Classification outcomes across neonatal developmental stages are summarized in Figure 15. The confusion matrix reveals strong discriminative capability, particularly for later monthly categories (5–9 months), where misclassifications are minimal. In contrast, early developmental intervals (0–30 days) show limited overlap with adjacent stages, which aligns with the gradual and subtle anatomical changes during the neonatal period.

A detailed overview of class-specific metrics is provided in Table 6. Most categories achieve accuracy levels above 98%, with precision and recall values consistently surpassing 90%. The best performance is observed for the 5-month (F1-score: 97.86%) and 4-month (F1-score: 95.94%) stages, whereas slightly reduced scores are noted for transitional classes such as 21–30 days (F1-score: 88.89%). These findings underscore the robustness of the proposed framework, highlighting the complementary value of integrating T1- and T2-weighted modalities for reliable characterization of neonatal brain development.

### 3.6. Performance on Combined T1 Female and Male Sequences

**Figure 16:**
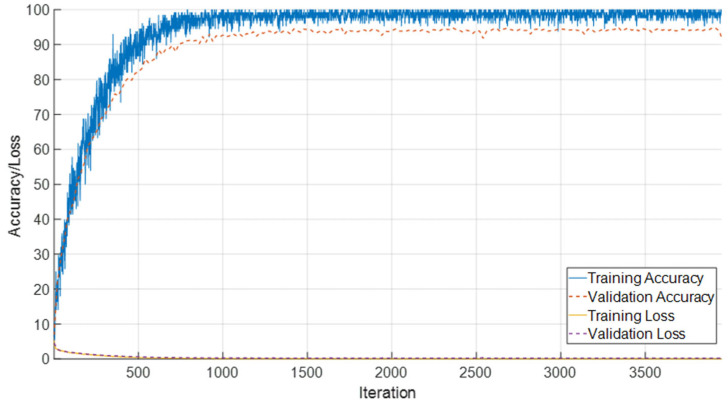
Training and validation accuracy and loss curves for the combined T1 female and male dataset with 28 classes.

In this section, the classification performance of the proposed framework on the combined T1-weighted female and male dataset, consisting of 28 developmental subgroups, is presented. The learning curves, as shown in Figure 16, demonstrate that the model achieved rapid convergence, with training and validation accuracies exceeding 95% after approximately 500 iterations and remaining stable thereafter. Both training and validation losses declined sharply during the early iterations and reached consistently low values, indicating effective learning without overfitting.

**Figure 17:**
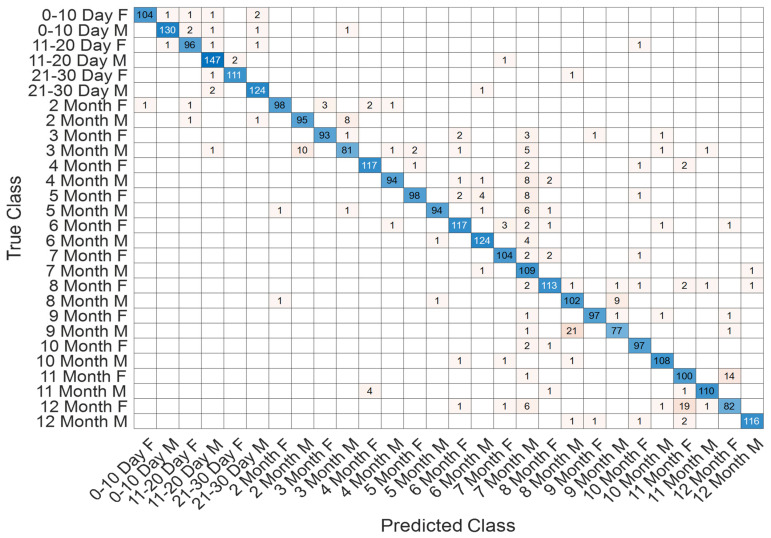
Confusion matrix showing classification outcomes across 28 age–sex subgroups in the T1 sequence.

The detailed classification outcomes are visualized in Figure 17, which depicts the confusion matrix across all 28 subgroups. The matrix is strongly diagonal, reflecting the high discriminative capacity of the model. Misclassifications were relatively infrequent and primarily observed between temporally adjacent classes, such as neighboring months, which can be attributed to the natural overlap in neurodevelopmental patterns during these stages. Importantly, early neonatal groups (0–10 days, 11–20 days, and 21–30 days) exhibited near-perfect recognition rates, highlighting the model’s robustness in detecting subtle maturational differences within the first month of life.

Comprehensive performance metrics for each subgroup are reported in Table 7. The results reveal consistently high accuracies, with most classes exceeding 99%. Precision, recall, and F1-scores were similarly strong across the majority of categories. While certain later stages, such as 7–8 months and 12 months, demonstrated slightly lower precision and recall, the overall performance remained robust, underscoring the model’s ability to capture fine-grained developmental trajectories in both sexes. Collectively, these findings confirm the effectiveness of the proposed approach for age- and sex-specific classification in neonatal brain development using T1-weighted MRI data.

**Figure 18:**
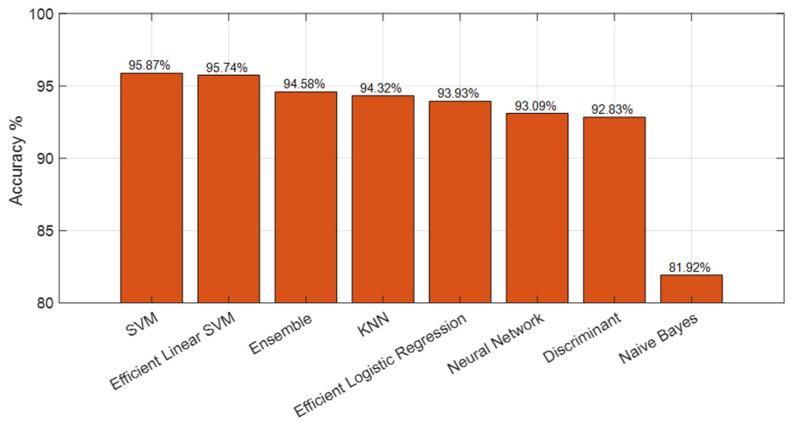
Ten-fold cross-validation results using classical machine learning classifiers applied to SEPoolConvNeXt-derived features from the T1 Female dataset.

To further evaluate the generalization capability of the proposed SEPoolConvNeXt framework and to examine the discriminative strength of the learned representations, a complementary experiment was conducted using traditional machine learning classifiers. Feature vectors extracted from the fully connected layer of the SEPoolConvNeXt model, trained on the T1 Female dataset, were used as inputs for classification under a ten-fold cross-validation protocol. As illustrated in Figure 18, the Support Vector Machine (SVM) and Efficient Linear SVM [42,43] classifiers achieved the highest accuracies, reaching 95.87% and 95.74%, respectively. Ensemble [44] and K-Nearest Neighbor (KNN) [45,46] classifiers followed closely with accuracies of 94.58% and 94.32%. Efficient Logistic Regression [47] and Neural Network [48] classifiers yielded accuracies of 93.93% and 93.09%, respectively, while the Discriminant classifier achieved 92.83%. The Naïve Bayes classifier produced the lowest performance, with an accuracy of 81.92%. These findings confirm that the features extracted by SEPoolConvNeXt possess high discriminative quality and generalize effectively across different classification paradigms. The consistent results across independent classifiers further demonstrate that the superior performance of the proposed model is not attributable to overfitting but reflects robust feature representation and generalization capability.

Overall, the experimental analyses conducted across T1- and T2-weighted sequences, stratified by sex and integrated across modalities, consistently demonstrated the robustness and generalization capability of the proposed SEPoolConvNeXt framework. The model achieved high accuracy, precision, recall, and F1-scores in nearly all developmental subgroups, with only minor reductions observed in certain intermediate and late monthly categories where biological overlap is inherently pronounced. The results further highlighted the capacity of the framework to adapt effectively across sex-specific variations and multimodal inputs, underscoring its potential as a reliable tool for fine-grained characterization of neonatal brain maturation. These findings collectively confirm the suitability of the proposed approach for automated age classification in early neurodevelopmental assessment and provide a solid foundation for subsequent clinical translation and application.

Table 8 presents the classification accuracies obtained through 10-fold cross-validation [49] using feature vectors extracted from the SEPoolConvNeXt model. The highest accuracy was achieved on the T2 Female sequence (96.03%), followed by the T1 Female (95.87%) and T1 Male (94.42%) datasets. The relatively lower performance on the T2 Male sequence (64.33%) likely reflects greater intra-class variability and contrast heterogeneity across male subjects. Combined evaluations (T1 + T2 Female and T1 Male + Female) demonstrated strong overall generalization, confirming that SEPoolConvNeXt-derived features are highly discriminative and robust across different modalities and sex-specific subgroups.

**Figure 19:**
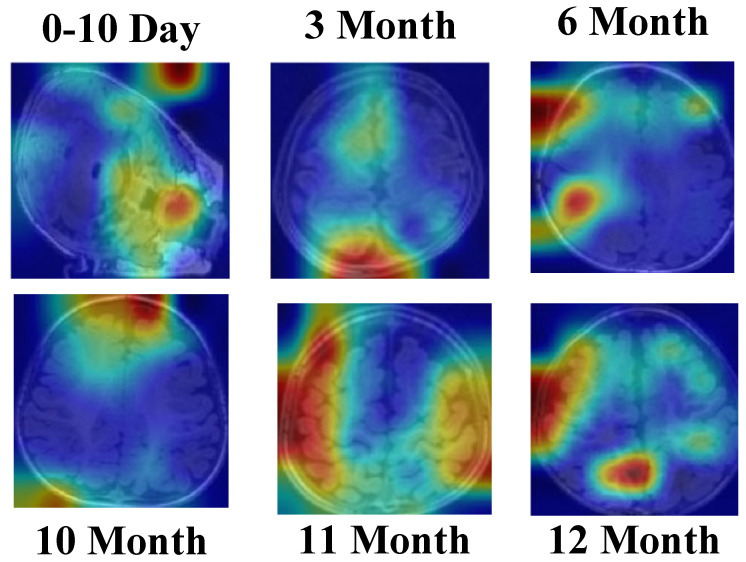
Grad-CAM [50,51] visualizations of the SEPoolConvNeXt model on the T1 Female dataset across representative developmental stages (0–10 days, 3, 6, 10, 11, and 12 months), illustrating the model’s attention to neuroanatomically relevant regions associated with progressive myelination and cortical maturation.

Figure 19 demonstrates that the SEPoolConvNeXt model consistently focuses on neuroanatomically meaningful regions such as the internal capsule, corpus callosum, and perirolandic cortex across different developmental stages. The gradual spatial shift of activation patterns reflects the normal progression of myelination and cortical maturation, supporting the biological plausibility and interpretability of the proposed framework.

## 4. Discussion

This study investigated automated classification of neonatal brain development using the proposed SEPoolConvNeXt model on the T1-weighted female dataset. To establish reference baselines, 19 widely used pre-trained CNN architectures were systematically evaluated under identical conditions. The results, summarized in Table 9, show that the accuracies of these networks ranged from 60.23% (NASNetMobile) to 78.31% (EfficientNetb0), with an overall mean of approximately 70.7%. These findings indicate that conventional transfer learning strategies from natural images achieve only moderate performance when applied to neonatal MRI classification.

The comparative evaluation of 22 deep learning architectures on the T1 Female dataset, including 19 conventional convolutional networks and three transformer-based or hybrid models (ViT, Swin Transformer, and ConvNeXt), provided a comprehensive benchmark for assessing the proposed SEPoolConvNeXt framework. Among the baseline models, ViT achieved the highest accuracy (82.76%), followed closely by ConvNeXt (82.18%), whereas EfficientNetb0 yielded the best performance among conventional CNNs with 78.31%. Deep residual and densely connected networks, such as ResNet101 and DenseNet201, achieved 75.08%, while classical models including VGG16, VGG19, and AlexNet reached approximately 72–73%. Lightweight models such as MobileNetV2, ShuffleNet, and SqueezeNet performed similarly (~71–73%), demonstrating that excessive parameter reduction reduces sensitivity to subtle developmental cues. Architectures primarily optimized for large-scale natural image recognition, including GoogLeNet, Inception variants, and NASNet models, performed less effectively (60–65%), highlighting their limited adaptability to neonatal MRI characterized by gradual and fine-grained anatomical contrast variations.

In contrast, SEPoolConvNeXt achieved accuracies consistently exceeding 95% across all datasets, with high precision, recall, F1-score, and AUC values, surpassing all pre-trained baselines by margins ranging between 17% and 35%. This substantial improvement results from the model’s domain-specific architectural design, which integrates grouped convolutions, residual pathways, and channel attention mechanisms to effectively capture the gradual signal transitions associated with early myelination and cortical maturation. Whereas pre-trained CNNs often misclassified adjacent developmental categories—such as between the third and fourth or the eleventh and twelfth months—SEPoolConvNeXt demonstrated greater robustness in distinguishing these subtle temporal transitions. The model’s capacity to recognize fine-grained structural and contrast variations confirms its suitability for accurate and biologically meaningful classification of neonatal brain maturation.

A notable strength of SEPoolConvNeXt is its computational efficiency, achieved with approximately 9.4 million trainable parameters. This compact design enables rapid inference and scalability, which are critical for clinical deployment where computational resources and time constraints are significant. The framework maintains a balance between model complexity and interpretability, positioning it as a practical solution for integration into radiological workflows. Despite these advantages, several methodological considerations warrant discussion. The comparative CNNs were employed primarily for feature extraction rather than full fine-tuning, which may have modestly limited their performance. Moreover, the analyses were conducted on two-dimensional MRI slices rather than three-dimensional volumetric data, restricting spatial continuity and inter-slice context. The dataset was also derived from a single clinical center, emphasizing the importance of future validation on multi-site, multi-scanner datasets to ensure generalizability and reproducibility.

Future research should address these aspects by extending SEPoolConvNeXt to 3D and multimodal (T1 + T2) configurations that can better capture complex neurodevelopmental patterns. Incorporating explainable AI methods such as Grad-CAM and SHAP will enhance interpretability and clinical confidence by highlighting neuroanatomically relevant activation regions. Further, longitudinal studies linking model-inferred developmental stages with neurocognitive outcomes will provide valuable insight into the predictive utility of MRI-derived biomarkers. From a translational perspective, the next stage of development should focus on creating a clinically integrated, PACS-compatible decision-support system capable of handling incomplete or motion-degraded scans. Such a system could also be extended to preterm and high-risk populations for early identification of delayed myelination or neurodevelopmental abnormalities.

Clinically, the implications of this study are substantial. Automated developmental staging using SEPoolConvNeXt provides an objective, standardized tool for assessing normative brain maturation and detecting deviations from typical trajectories. By minimizing observer variability, the framework enhances diagnostic consistency and complements expert radiological interpretation with quantitative biomarkers. Its demonstrated ability to capture age- and sex-specific developmental differences supports its potential use in early detection of atypical brain development, particularly in high-risk neonates such as preterm infants. In longitudinal applications, SEPoolConvNeXt may further contribute to individualized monitoring, early intervention planning, and improved neurodevelopmental outcomes during the critical first year of life.

In summary, the comparative evaluation confirmed that generic pre-trained CNNs offer limited accuracy on neonatal MRI, whereas SEPoolConvNeXt provides promising performance as a technical prototype. By aligning architectural innovations with biological characteristics of early brain development, the proposed model establishes a potential foundation for future clinical translation in neonatal neuroimaging.

## 5. Conclusions

This study introduced SEPoolConvNeXt, a domain-specific deep learning framework for automated classification of neonatal brain development across age- and sex-stratified subgroups using T1- and T2-weighted MRI sequences. The proposed model consistently achieved state-of-the-art performance, with accuracies exceeding 95% across nearly all developmental categories, substantially outperforming 22 benchmark architectures—including 19 conventional CNNs and three contemporary transformer-based or hybrid models (ViT, Swin Transformer, and ConvNeXt). These results underscore the limited transferability of general-purpose image networks to neonatal MRI and highlight the advantages of a biologically tailored design.

SEPoolConvNeXt’s architecture, which combines grouped convolutions, residual pathways, and channel attention mechanisms, effectively captures the gradual contrast transitions and fine-grained anatomical variations characteristic of early brain maturation. The model achieved high precision, recall, F1-score, and AUC values while maintaining computational efficiency (~9.4 M parameters), confirming its suitability for real-world clinical deployment. Evaluations across T1-weighted, T2-weighted, and combined datasets further demonstrated its robustness and generalizability, with most subgroups achieving accuracies above 98%.

Clinically, SEPoolConvNeXt provides an objective and standardized tool for assessing normative brain maturation, complementing expert radiological evaluation with quantitative biomarkers. Its capability to detect subtle, sex- and age-specific developmental differences positions it as a valuable aid in the early identification of atypical trajectories and neurodevelopmental delays, particularly in preterm or high-risk neonates. Longitudinal application of this framework may enhance early intervention planning and support continuous neurodevelopmental monitoring during infancy.

In summary, SEPoolConvNeXt represents a robust, efficient, and biologically aligned solution for neonatal brain maturation assessment. By integrating architectural innovation with domain-specific insight, it establishes a strong foundation for reliable AI-assisted neurodevelopmental evaluation and future clinical translation.

## Figures and Tables

**Table 1:** Distribution of training and test samples across T1 and T2 sequences.

Group	T1 Male Sequence			T1 Female Sequence			T2 Male Sequence			T2 Female Sequence		
	Total Images	Train	Test	Total Images	Train	Test	Total Images	Train	Test	Total Images	Train	Test
0–10 Day	675	540	135	543	434	109	449	359	90	374	299	75
11–20 Day	752	602	150	499	399	100	538	430	108	378	302	76
21–30 Day	637	510	127	566	453	113	294	235	59	246	197	49
2 Month	523	418	105	529	423	106	507	406	101	520	416	104
3 Month	516	413	103	505	404	101	485	388	97	478	382	96
4 Month	529	423	106	617	494	123	557	446	111	505	404	101
5 Month	519	415	104	567	454	113	502	402	100	503	402	101
6 Month	644	515	129	630	504	126	510	408	102	506	405	101
7 Month	556	445	111	547	438	109	504	403	101	501	401	100
8 Month	566	453	113	610	488	122	555	444	111	500	400	100
9 Month	499	399	100	507	406	101	540	432	108	517	414	103
10 Month	554	443	111	501	401	100	564	451	113	513	410	103
11 Month	581	465	116	576	461	115	507	406	101	501	401	100
12 Month	603	482	121	557	446	111	554	443	111	500	400	100
Total	8154	6523	1631	7754	6205	1549	7066	5653	1413	6542	5233	1309

**Table 2:** Class-wise evaluation metrics including accuracy, precision, recall, specificity, and F1-score for the T1 Female dataset.

Class	TP	FP	FN	TN	Accuracy	Precision	Recall	Specificity	F1-Score
0–10 Day	108	0	1	1440	99.94%	100.00%	99.08%	100.00%	99.54%
11–20 Day	100	1	0	1448	99.94%	99.01%	100.00%	99.93%	99.50%
21–30 Day	112	0	1	1436	99.94%	100.00%	99.12%	100.00%	99.56%
2 Month	106	1	0	1442	99.94%	99.07%	100.00%	99.93%	99.53%
3 Month	96	1	5	1447	99.61%	98.97%	95.05%	99.93%	96.97%
4 Month	121	3	2	1423	99.68%	97.58%	98.37%	99.79%	97.98%
5 Month	110	8	3	1428	99.29%	93.22%	97.35%	99.44%	95.24%
6 Month	124	7	2	1416	99.42%	94.66%	98.41%	99.51%	96.50%
7 Month	103	3	6	1437	99.42%	97.17%	94.50%	99.79%	95.81%
8 Month	113	4	9	1423	99.16%	96.58%	92.62%	99.72%	94.56%
9 Month	100	0	1	1448	99.94%	100.00%	99.01%	100.00%	99.50%
10 Month	97	2	3	1447	99.68%	97.98%	97.00%	99.86%	97.49%
11 Month	106	21	9	1413	98.06%	83.46%	92.17%	98.54%	87.60%
12 Month	92	10	19	1428	98.13%	90.20%	82.88%	99.30%	86.38%

**Table 3:** Class-wise performance metrics (Accuracy, Precision, Recall, Specificity, and F1-score) of SEPoolConvNeXt on the T1 Male sequence.

Class	TP	FP	FN	TN	Accuracy	Precision	Recall	Specificity	F1-Score
0–10 Day	133	2	2	1494	99.75%	98.52%	98.52%	99.87%	98.52%
11–20 Day	138	1	12	1480	99.20%	99.28%	92.00%	99.93%	95.50%
21–30 Day	126	10	1	1494	99.33%	92.65%	99.21%	99.34%	95.82%
2 Month	89	10	16	1516	98.41%	89.90%	84.76%	99.34%	87.25%
3 Month	93	21	10	1507	98.10%	81.58%	90.29%	98.63%	85.71%
4 Month	104	2	2	1523	99.75%	98.11%	98.11%	99.87%	98.11%
5 Month	102	3	2	1524	99.69%	97.14%	98.08%	99.80%	97.61%
6 Month	125	1	4	1501	99.69%	99.21%	96.90%	99.93%	98.04%
7 Month	108	4	3	1516	99.57%	96.43%	97.30%	99.74%	96.86%
8 Month	100	18	13	1500	98.10%	84.75%	88.50%	98.81%	86.58%
9 Month	83	12	17	1519	98.22%	87.37%	83.00%	99.22%	85.13%
10 Month	110	2	1	1518	99.82%	98.21%	99.10%	99.87%	98.65%
11 Month	115	1	1	1514	99.88%	99.14%	99.14%	99.93%	99.14%
12 Month	118	0	3	1510	99.82%	100.00%	97.52%	100.00%	98.74%

**Table 4:** Class-wise performance metrics of the proposed SEPoolConvNeXt model for the T2 Female sequence.

Class	TP	FP	FN	TN	Accuracy	Precision	Recall	Specificity	F1-Score
0–10 Day	75	0	0	1234	100.00%	100.00%	100.00%	100.00%	100.00%
11–20 Day	100	0	3	1206	99.77%	100.00%	97.09%	100.00%	98.52%
21–30 Day	99	4	1	1205	99.62%	96.12%	99.00%	99.67%	97.54%
2 Month	75	0	1	1233	99.92%	100.00%	98.68%	100.00%	99.34%
3 Month	96	3	4	1206	99.47%	96.97%	96.00%	99.75%	96.48%
4 Month	104	2	0	1203	99.85%	98.11%	100.00%	99.83%	99.05%
5 Month	49	0	0	1260	100.00%	100.00%	100.00%	100.00%	100.00%
6 Month	95	1	1	1212	99.85%	98.96%	98.96%	99.92%	98.96%
7 Month	100	0	1	1208	99.92%	100.00%	99.01%	100.00%	99.50%
8 Month	100	2	1	1206	99.77%	98.04%	99.01%	99.83%	98.52%
9 Month	99	0	2	1208	99.85%	100.00%	98.02%	100.00%	99.00%
10 Month	100	4	0	1205	99.69%	96.15%	100.00%	99.67%	98.04%
11 Month	98	1	2	1208	99.77%	98.99%	98.00%	99.92%	98.49%
12 Month	102	0	1	1206	99.92%	100.00%	99.03%	100.00%	99.51%

**Table 5:** Quantitative performance metrics (accuracy, precision, recall, specificity, and F1-score) of the proposed framework on the T2 male sequence.

Class	TP	FP	FN	TN	Accuracy	Precision	Recall	Specificity	F1-Score
0–10 Day	75	6	15	1317	98.51%	92.59%	83.33%	99.55%	87.72%
11–20 Day	84	20	24	1285	96.89%	80.77%	77.78%	98.47%	79.25%
21–30 Day	42	24	17	1330	97.10%	63.64%	71.19%	98.23%	67.20%
2 Month	78	40	23	1272	95.54%	66.10%	77.23%	96.95%	71.23%
3 Month	67	22	30	1294	96.32%	75.28%	69.07%	98.33%	72.04%
4 Month	82	24	29	1278	96.25%	77.36%	73.87%	98.16%	75.58%
5 Month	64	58	36	1255	93.35%	52.46%	64.00%	95.58%	57.66%
6 Month	69	34	33	1277	95.26%	66.99%	67.65%	97.41%	67.32%
7 Month	61	41	40	1271	94.27%	59.80%	60.40%	96.88%	60.10%
8 Month	57	54	54	1248	92.36%	51.35%	51.35%	95.85%	51.35%
9 Month	61	50	47	1255	93.14%	54.95%	56.48%	96.17%	55.71%
10 Month	73	50	40	1250	93.63%	59.35%	64.60%	96.15%	61.86%
11 Month	53	21	48	1291	95.12%	71.62%	52.48%	98.40%	60.57%
12 Month	70	33	41	1269	94.76%	67.96%	63.06%	97.47%	65.42%

**Table 6:** Classification metrics (Accuracy, Precision, Recall, Specificity, and F1-score) for each developmental stage using the combined T1 and T2 female sequences.

Class	TP	FP	FN	TN	Accuracy	Precision	Recall	Specificity	F1-Score
0–10 Day	183	5	0	2670	99.83%	97.34%	100.00%	99.81%	98.65%
11–20 Day	190	12	13	2643	99.13%	94.06%	93.60%	99.55%	93.83%
21–30 Day	188	20	27	2623	98.36%	90.38%	87.44%	99.24%	88.89%
2 Month	165	4	10	2679	99.51%	97.63%	94.29%	99.85%	95.93%
3 Month	192	26	19	2621	98.43%	88.07%	91.00%	99.02%	89.51%
4 Month	201	8	9	2640	99.41%	96.17%	95.71%	99.70%	95.94%
5 Month	160	5	2	2691	99.76%	96.97%	98.77%	99.81%	97.86%
6 Month	181	8	16	2653	99.16%	95.77%	91.88%	99.70%	93.78%
7 Month	209	15	15	2619	98.95%	93.30%	93.30%	99.43%	93.30%
8 Month	199	22	15	2622	98.71%	90.05%	92.99%	99.17%	91.49%
9 Month	213	12	14	2619	99.09%	94.67%	93.83%	99.54%	94.25%
10 Month	196	15	14	2633	98.99%	92.89%	93.33%	99.43%	93.11%
11 Month	206	9	16	2627	99.13%	95.81%	92.79%	99.66%	94.28%
12 Month	196	18	9	2635	99.06%	91.59%	95.61%	99.32%	93.56%

**Table 7:** Classification performance metrics for 28 subgroups in the T1 female and male dataset.

Class	TP	FP	FN	TN	Accuracy	Precision	Recall	Specificity	F1-Score
0–10 Day F	104	1	5	3070	99.81%	99.05%	95.41%	99.97%	97.20%
0–10 Day M	130	2	5	3043	99.78%	98.48%	96.30%	99.93%	97.38%
11–20 Day F	96	5	4	3075	99.72%	95.05%	96.00%	99.84%	95.52%
11–20 Day M	147	7	3	3023	99.69%	95.45%	98.00%	99.77%	96.71%
21–30 Day F	111	2	2	3065	99.87%	98.23%	98.23%	99.93%	98.23%
21–30 Day M	124	5	3	3048	99.75%	96.12%	97.64%	99.84%	96.88%
2 Month F	98	2	8	3072	99.69%	98.00%	92.45%	99.93%	95.15%
2 Month M	95	10	10	3065	99.37%	90.48%	90.48%	99.67%	90.48%
3 Month F	93	3	8	3076	99.65%	96.88%	92.08%	99.90%	94.42%
3 Month M	81	11	22	3066	98.96%	88.04%	78.64%	99.64%	83.08%
4 Month F	117	6	6	3051	99.62%	95.12%	95.12%	99.80%	95.12%
4 Month M	94	3	12	3071	99.53%	96.91%	88.68%	99.90%	92.61%
5 Month F	98	3	15	3064	99.43%	97.03%	86.73%	99.90%	91.59%
5 Month M	94	2	10	3074	99.62%	97.92%	90.38%	99.93%	94.00%
6 Month F	117	8	9	3046	99.47%	93.60%	92.86%	99.74%	93.23%
6 Month M	124	8	5	3043	99.59%	93.94%	96.12%	99.74%	95.02%
7 Month F	104	6	5	3065	99.65%	94.55%	95.41%	99.80%	94.98%
7 Month M	109	53	2	3016	98.27%	67.28%	98.20%	98.27%	79.85%
8 Month F	113	8	9	3050	99.47%	93.39%	92.62%	99.74%	93.00%
8 Month M	102	25	11	3042	98.87%	80.31%	90.27%	99.18%	85.00%
9 Month F	97	2	4	3077	99.81%	97.98%	96.04%	99.94%	97.00%
9 Month M	77	11	23	3069	98.93%	87.50%	77.00%	99.64%	81.91%
10 Month F	97	6	3	3074	99.72%	94.17%	97.00%	99.81%	95.57%
10 Month M	108	5	3	3064	99.75%	95.58%	97.30%	99.84%	96.43%
11 Month F	100	26	15	3039	98.71%	79.37%	86.96%	99.15%	82.99%
11 Month M	110	3	6	3061	99.72%	97.35%	94.83%	99.90%	96.07%
12 Month F	82	17	29	3052	98.55%	82.83%	73.87%	99.45%	78.10%
12 Month M	116	2	5	3057	99.78%	98.31%	95.87%	99.93%	97.07%

**Table 8:** Cross-validation performance of SEPoolConvNeXt-based features using multiple classifiers across different MRI sequences.

Sequences	Accuracy (%)	Precision (%)	Recall (%)	F1 Score (%)
T1 Female Sequences	95.87	95.21	95.17	95.18
T1 Male Sequences	94.42	94.29	94.30	94.29
T2 Female Sequences	96.03	96.90	96.54	96.70
T2 Male Sequences	64.33	65.60	65.21	65.35
Combined T1 and T2 Female Sequences	92.30	91.79	91.74	91.76
T1 Female and Male Sequences	93.43	93.58	93.41	93.46

**Table 9:** Comparative performance of 22 deep learning architectures on the T1 Female dataset using multiple metrics.

Number	Network	Accuracy (%)	Precision (%)	Recall (%)	F1-Score (%)
1	Efficientnetb0 [52]	78.31	77.42	78.07	77.74
2	Resnet101 [53]	75.08	74.65	74.93	74.79
3	Densenet201 [54]	75.08	74.85	74.66	74.75
4	Resnet50 [53]	74.50	74.23	73.97	74.10
5	Darknet53 [55]	74.50	74.18	73.94	74.06
6	Vgg19 [56]	73.08	72.54	72.89	72.71
7	Squeezenet [57]	72.82	72.13	72.57	72.35
8	Alexnet [58]	72.50	72.10	71.88	71.99
9	Vgg16 [56]	72.42	71.90	72.03	71.96
10	Xception [59]	72.05	71.54	71.63	71.58
11	Mobilenetv2 [60]	71.78	71.46	71.38	71.42
12	Shufflenet [61]	71.72	71.35	71.26	71.30
13	Resnet18 [53]	71.34	70.90	70.81	70.85
14	Darknet19 [55]	70.88	70.57	70.61	70.59
15	Inceptionresnetv2 [62]	69.39	69.06	68.98	69.02
16	Inceptionv3 [63]	65.27	64.88	64.75	64.81
17	Nasnetlarge [64]	64.16	63.92	63.78	63.85
18	Googlenet [65]	63.51	63.14	63.20	63.17
19	Nasnetmobile [64]	60.23	59.88	59.74	59.81
20	VİT(Vision Transformer) [66]	82.76	82.58	82.41	82.49
21	Swin Transformer [67]	77.86	77.43	77.50	77.46
22	ConvNeXt [68]	82.18	81.95	81.76	81.85

## Data Availability

The dataset can be downloaded at https://www.kaggle.com/datasets/buraktaci/neonatal-brain-development-mri (accessed on 13 October 2025).
